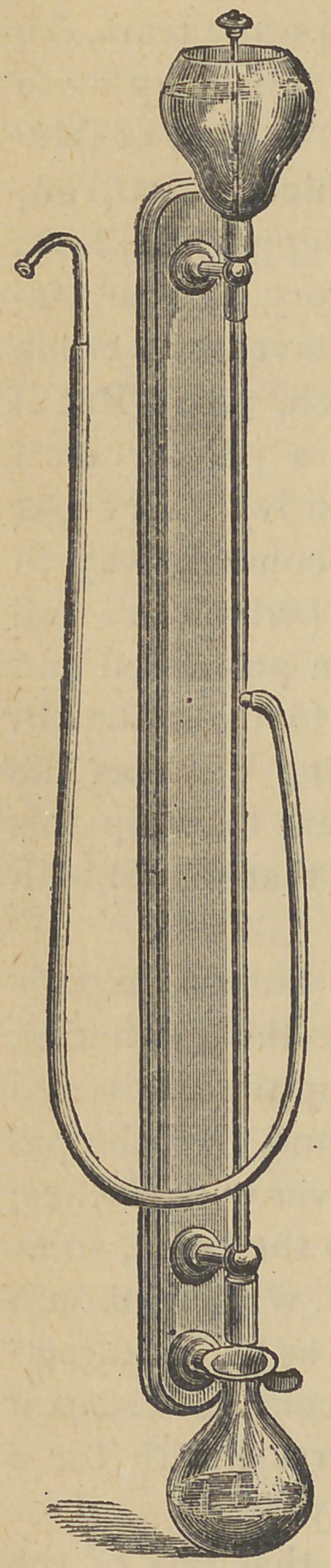# Editorial

**Published:** 1876-10

**Authors:** 


### Editorial.

### BAD TASTE.

In speaking of dental societies Dr. Horne says: "One who is a Sir Oracle to the circle of his local admirers, and comes in all the glory of delegateship to the annual reunion at Niagara, or Saratoga, or elsewhere, is soon toned down into a wholesome perception of the smallness of the horizon which his eye has been accustomed to scan, and finds that there are more things in heaven and earth than were dreamed of in his philosophy. He goes home a wiser and a better man; and his brethren of the Mad River Valley, or the Thunder and Lightning Hills, learn, in their turn, that there are other worlds than ours."

Such is the paragraph, and it may be fine writing; but the bad taste consists in the hint that all the stupid, narrow minded, mock Sir Oracle delegates to the American Dental Association come from two societies, and while one of them is concealed by a *nom de plume*, the other is named in full, the "Mad River Valley" society. The correspondent does himself injustice by such an ill-mannered fling at an active and honorable local society. He might lead some to suspect that he is the "*unknown*" writer named "*Snarler*" who, a few years ago was hired to blackguard a member of the Mad River Valley Society supposed to be dying. Such an inference is not unnatural, for while sinners can forgive those who have injured them, none but a saint can forgive those whom he has wronged.

What has the little society done that it must be anathematized by a bull from Rome? It has furnished presidents for the American Dental Association, about one fourth of the years of its existence, the present occupant of the presidency being one of its most active members. It includes in its membership a larger per cent of recognized authors and teachers than any other local society, perhaps. But, if all this were not so, the Roman sneer is in very bad taste.

But prophecy must be fulfilled. The prophet Daniel, in his great vision of the future, speaks of its events as already past, and tells us "there came up among them another little horn." Is this he that should come, or look we for another?

Why not send this to the *Cosmos*? says one. The REGISTER is *read* by the members of "Mad River." W.

### MAD RIVER VALLEY DENTAL ASSOCIATION

The Semi-Annual Meeting of the Mad River Valley Dental Association will be held in Xenia, Ohio, on Tuesday, October 3d, 1876, beginning at ten o'clock a. m.

TOPICS FOR DISCUSSION.

1. Sensitive Dentine; how may it be overcome?
2. Idiosyncrasies of the Patient to be considered as to success or failure in filling Teeth.
3. What success are we meeting with in preserving exposed Nerves?
4. Our Diet in relation to the Teeth.

5. Derangement of the organism consequent upon diseased Teeth.

6. Relative merits of Rubber and Celluloid as Bases for Artificial Teeth.

ESSAYISTS.

Dr. C. Bradley, Dr. E. F. Sample, Dr. A. T. Whiteside, Dr. C. R. Sabin, Dr. J. A. Stipp, Dr. E. G. Betty.

EXECUTIVE COMMITTEE.

Drs. S. B. Tizzard, E. F. Sample, J. H. Paine.

Geo. W. Keely, President.

J. L. Zell, Secretary.

### CONNECTICUT VALLEY DENTAL SOCIETY.

The fourteenth annual Meeting of the Connecticut Valley Dental Society, will be held at Haynes' Hotel, Springfield, Mass., commencing on Tuesday, October 17th, 1876, at ten o'clock, a. m. H. F. Bishop, Worcester, Mass., President. L. C. Taylor, J. N. Davenport and Lester Noble. Executive Committee.

SUBJECTS AND ESSAYISTS.

Professional Deportment, Dr. H. M. Miller, Westfield, Mass.

How shall children's teeth, between the ages of twelve and eighteen be treated? Dr. L. D. Shepard, Boston, Mass.

Is there a time when a tooth, in an abscessed condition, should not be extracted? Dr. F. Searle, Springfield, Mass.

Exostosis, its Diagnosis and Treatment, Dr. J. M. Riggs, Hartford, Conn.

Is Irregular Dentition Hereditary? Prof. C. A. Brackett Newport, R. I.

Health of the Dentist, Dr. W. W. Sheffield, New London, Conn.

Spontaneous Death of Pulps, Dr. L. Noble, Springfield, Mass.

Miscellaneous Subjects.

The Executive Committee hereby extend a cordial invitation, not only to every member of the Society, but to the profession at large, to be present at this meeting; and in order that this may be a profitable and successful one, it is incumbent upon every one who proposes to attend to regard this as a mutual school of the profession, where all are teachers and all learners. Assembling in this spirit, having previously looked over the list of topics presented, and having selected one, or more, upon which thought and study has been bestowed, each one may thus come prepared to promptly discuss the subjects, as presented, and express his thoughts and opinions in a concise and lucid manner; thus ensuring a lively, interesting and profitable session, making our remarks worthy of consideration.

Members and others, who may have pet instruments, interesting cases in models, or drawings, or instruments of new patterns, etc., are requested to bring them.

Special arrangements for accomdations, at the Haynes House, may be made at from $2.50 to $3.00 per day.

A resolution will be offered at the first session, giving the Ex. Com. instructions to employ a "short hand" reporter for the purpose of securing a full and complete report of this and future meetings, for publication.

Members of the medical profession are very cordially invited to be present at the evening session and participate in the discussions.

It is earnestly desired that each member will feel it incumbent upon himself to be promptly on hand at the opening of the first session, as important business occurs.

C. T. Stockwell, Sec'y.

Springfield, Mass., Sept. 6, 1876.

### SPECIAL MEETING.

The Michigan Dental Association will meet in Special Session, on the 10th day of October, 1876, in the city of Detroit, at the Russell House. The Association will be called to order at ten o'clock a. m.

The special business of this session will be to hear and act upon the report of the committee of sixteen, on State Association Charter, and Dental Law. Also, the following important papers:

"Dental Legislation and its Necessity," by E. S, Holmes, D. D. S.

"Alveolar Abscess," by W. H. Jackson, D. D. S.

"Sensitive Dentine," by I. Douglass, D. D. S.

"Reproduction of Tissue," by D. D. Hawxhurst, D. D. S., will be presented for the consideration of the Association.

The importance of this meeting demands the attention of every member of the association, and every other progressive dentist in the State. Other business will undoubtedly be brought up, which you should be present to attend to.

E. S. Holmes,
Secretary Michigan Dental Association.

### "FRAGMENTARY CLIPPINGS."

Under this heading, on page 289, of this volume of the Register, Prof. Cutler unintentionally credits the writer of this with a quotation from a communication in which the writer quoted from memory.

Prof. C. sneers at the minute size of a supposed battery in the mouth; yet small as it is, it would send a message across the Atlantic.

But this is a small matter. Farther down the page Prof. C. becomes enclouded in a fog more dense than I supposed to exist in the Mississippi Valley. He says: "Admitting, for argument's sake, again, that ammonia and nitric acid are both formed in the mouth simultaneously, the result of this double binary process must necessarily be nitrate of ammonia formed instantaneously, and no free acid left."

And he thinks I must have overlooked this fact. Not so But has he overlooked the fact that the nitrates are all soluble, and that, therefore, nitric acid acts on all the bases presented to it, which, in the case supposed, includes the lime of the teeth?

I feel confident that Prof. C. has not understood my views in relation to dental caries, at all, and feel quite as confident he will endorse them when he makes a thorough examination of the subject. W.

### FIAT JUSTITIA.

On page 410, vol. xviii, of the "Dental Cosmos," Dr. Horne, of Rome, says:

"The Code of Ethics of the American Dental Association, was compiled and its adoption secured chiefly through the efforts of Dr. John Allen."

On the same page he says farther:

"The precepts enjoined in relation to professional character and deportment, are such as must commend themselves to every dentist possessed of a proper idea of the duties and dignity of his position." Which is highly complimentary to the code, and probably expresses the general sentiment of the profession in reference to it.

But we believe Dr. H. slightly misapprehends the history of the Code of Ethics. At the meeting of the association, held in Boston, 1866, Dr. Watt made the motion for a committee to report a code of ethics. He was appointed chairman, with Drs. Allen and McQuillen as co-members. Dr. McQ. did not meet with the committee, being deterred by other duties, as he explained on the floor of the association. Dr. Allen had read a paper on the subject at the previous annual meeting, which was before the committee in manuscript but not used. In short, the code of the American Dental Association is a revised copy of the code of the Ohio State Dental Society, prepared for that society by a committee composed of Drs. Watt and Taft.

As the motion to appoint the committee was made by Dr. W., the report made by him, read to the association, explained, in answer to enquiries, and was defended against adverse criticisms mainly by him, it seems hardly correct to say that the code of ethics "was compiled and its adoption secured chiefly through the efforts of Dr. John Allen."

### CELSUS.

Toward the latter end of the first century, people had tooth ache just as they have to-day, but their method of treating it presents a curious contrast to that of the present, which appears in the following extract from Celsus' book on medicine, and containing all the information he has to give upon the subject. It will be observed that things have changed since this work was written, and that the practical treatment of tooth ache now might be stated in fewer words.

"ON THE TOOTH ACHE."

"In the tooth ache, a disorder that may justly be ranked even amongst the greatest torments, the use of wine must be entirely forbiden, and at first a total abstinence from food must be observed; afterwards it may be taken sparingly, but soft, lest the teeth be irritated by chewing. Then externally, by means of a sponge, the steam of hot water is to be applied, and a cerate made of cyprine or iris oil spread upon wool, and the head must also be covered. But if the pain be more severe, a clyster is useful with hot cataplasms applied to the cheeks, as also some medicinal hot liquor held in the mouth, and frequently changed; for which purpose is used a decoction of cinquefoil root in diluted wine, and henbane root, either in vinegar and water, or diluted wine, with the addition of a little salt to either of them; and poppy heads not over dry, and mandrake root prepared in the same manner. But in these three care must be taken not to swallow what is in the mouth. The bark of the root of the white poplar boiled in diluted wine does very well for this purpose; or hartshorn shavings in vinegar, and catmint with teda, and a mellow fig; also a mellow fig, either in mulse or in vinegar and honey, and when the fig is dissolved by boiling, the liquor is strained. A probe, also, wrapt up in wool, is dipped in hot oil and used to touch the tooth itself. Moreover, something like cataplasms are put into the tooth. For which end the minor part of an acid and dry pomegranate is powdered with an equal quantity of galls and pine bark, and with these is mixed minium which being powdered are brought to a consistence with rain water; or panaces, poppy tears, hog's fennel, stavesacre without its seeds, powered in equal proportions; or three parts of galbanium and a fourth of poppy tears. Whatever is applied to the teeth, a cerate such as is directed above, ought, nevertheless to be kept upon the cheek and covered with wool. Some also bruise and spread upon linen, myrrh, cardomens, of each p,i; saffron pellitory, figs, pepper, each, p,iv; mustard p,viii, and apply this to the arm of that side which the painful tooth is; if it be in the upper jaw, in the part next the scapula; if in the lower, on that next the breast; and this relieves the pain. And when it has given ease, it must be immediately taken away, Now if the tooth be spoilt, we need not be hasty in extracting it, unless there be a necessity for it; but in such case, to all the fomentations directed before must be added some stronger compositions to ease the pain; such as that which contains of poppy tears, p,i, pepper, ii, sory, p,x; these are powdered and mixed up with galbanum, and put round the affected tooth; or that of Menemachus, principally for double teeth; in which are saffron, p,i, cardomons, soot of frankincense, figs, pepper, pelitory, each, p,iv; mustard, p,viii. Some mix of pellitory, pepper, elaterium, each, p,i; scissile alum, poppy tears, stavesacre, crude sulphur, bitumen, bay berries, mustard. of each, p,ii. But if the pain make it necessary to take it out, a pepper corn stript of its bark, and in the same manner, an ivy berry put into its opening splits the tooth so that it comes away in scales. The prickle of the planus fish also (which we call pastinaca, the Greeks trygon) is toasted, then powdered and mixed with resin, which being put round the tooth, loosens it.

Scissile alum likewise put into the opening disposes the tooth to come away. But it is more expedient to wrap this in a little wool, and then put it in; because in that way it both preserves the tooth and eases the pain.

These are the prescriptions of physicians, but the experience of our peasants has discovered, that for the toothache, the herb horse mint ought to be pulled up by the roots and put into a basin and water infused upon it; and that the patient should sit down close by it, covered all over with clothes; and then red hot flints are to be thrown into the basin, so as to be covered with the water; and the patient, with his mouth open, must receive the vapor, close wrapt up as before directed. For both a plentiful sweat follows and a continued stream of rheum runs from the mouth, which secures health for a pretty long time, and frequently for a whole year,"

In publishing the above it is not expected that any of the brethren will adopt the practice, but it is well enough to know what was going on about the time of Celsus

### A NEW SALIVA EXTRACTOR.

This is an appliance devised and recently completed by Dr. Geo. B. Snow, of Buffalo. It is an excellent instrument, one that accomplishes its work most effectually. It is portable, can be placed wherever it is desired. It is uniform in its action, and it is perfectly under the control of the operator or assistant.

The apparatus consists of a receiver, from which water escapes, drop by drop, downward through a tube of small bore. Capillary attraction causes the water to completly fill the bore of the tube, and to act as a piston. A peculiar arrangement at the top of the tube produces an alternation of the supply from water to air. By this means the air is removed and a partial vacuum effected of a larger tube which surrounds the small one first mentioned, as a casing, and the saliva is drawn into the large tube from the mouth and falls into its bottom where it escapes. The bottom of the large tube is open, but it is rendered air tight by being *trapped* by water contained in the cup at its lower end.

The principle of its action partakes somewhat of that of both the syphon and Giffard Injector.

Every office should be supplied with a saliva extractor, and where there is not running water, this one will certainly supply a great want.

The accompanying cut gives a correct idea of the eneral form of the instrument and of its parts.

## Figures and Tables

**Figure f1:**